# Assessment of removal and adsorption enhancement of high-flux hemodialyzers in convective therapies by a novel in vitro uremic matrix

**DOI:** 10.1038/s41598-020-74528-5

**Published:** 2020-10-15

**Authors:** Miquel Gomez, Elisenda Bañon-Maneus, Marta Arias-Guillén, Francisco Maduell

**Affiliations:** 1 Laboratori Experimental de Nefrologia i Trasplantament (LENIT), Fundació Clínic per la Recerca Biomédica (FCRB), Hospital Clínic de Barcelona, Barcelona, Spain; 2 Red de Investigación Renal (REDINREN), Madrid, Spain; 3 Department of Nephrology, Hospital Clínic de Barcelona, Barcelona, Spain

**Keywords:** Nephrology, Renal replacement therapy

## Abstract

Adsorption properties of hemodialyzers are traditionally retrieved from diffusive treatments and mainly focused on inflammatory markers and plasma proteins. The possible depurative enhancement of middle and high molecular weight solutes, as well as protein-bound uremic toxins by adsorption in convective treatments, is not yet reported. We used discarded plasma exchanges from uremic patients and out-of-date erythrocytes as a novel in vitro uremic precursor matrix to assess removal and adsorption patterns of distinct material and structure but similar surface hemodialyzers in hemodialysis and on-line hemodiafiltration treatments. We further related the obtained results to the possible underlying membrane pore blocking mechanisms. Convection improved removal but slightly enhanced adsorption in the cellulosic and synthetic dialyzers tested. The polymethylmethacrylate hemodialyzer obtained the highest extracted ($M_{ext}$) and adsorbed ($M_{ads}$) mass values when submitted to hemodiafiltration for all molecules analyzed including albumin ($M_{ext}=15.8\pm 3.9$ g, $M_{ads}=44.3\pm 11.5$ mg), whereas the polyamide membrane obtained substantial lower results even for this molecule ($M_{ext}=2.2\pm 1.2$ g, $M_{ads}=4.2\pm 0.7$ mg) under the same treatment parameters. Hemodiafiltration in symmetric and enlarged pore hemodialyzers enhances removal and adsorption by internal pore deposition (intermediate pore-blocking) for middle and high molecular weight toxins but leads to substantial and deleterious albumin depuration.

## Introduction

Hemodialysis is a renal replacement therapy^1^ which exploits the properties of a semipermeable membrane (or hemodialyzer) to maintain patient over-hydration, assess electrolyte balance and attain tolerable levels of uremic surrogates^2^ through diffusive (high-flux hemodialysis, HD) or convective enhanced (hemodiafiltration, HDF) blood cleaning processes. Moreover, the interaction of blood with a particular exogenous material^3,4^ (cellulosic or synthetic) and structure (symmetric or asymmetric), like the hemodialysis hollow fiber capillary, may lead to adsorption^5,6^. The precise mechanisms of adsorption in hemodialysis are not fully understood^7^ but are likely related to the interaction of solutes to the inner surface and the porous frame of the membrane^8,9^. Up-to-date, the adsorption properties of distinct available hemodialyzers have been obtained mainly from HD treatments with very few exceptions^10^ and focused on cytokines and interleukins, $\beta _{2}$-microglobulin, TNF-$\alpha$ and essential plasma proteins such as albumin^11–15^. Thus, there is a lack of information regarding how convection and hemoconcentration in HDF and hydrodynamic phenomena, such as cell-free zone from the Fahraeus–Lindqvist effect^16^, may compress middle and high molecular weight (MW) uremic toxins toward the capillary inner wall and lead the formation of concentration polarization and a protein cake layer^17^. The promotion of adsorption by HDF may reduce hemodialyzer performance due to fouling as described by four possible blocking regimes in membrane filtration^18,19^ (i.e., complete, standard, intermediate and cake filtration) depending on the solute-to-pore preferential deposition sites. These models are, nevertheless, scantly implemented in hemodialysis^20,21^. The evaluation of the depurative enhancement by adsorption in HDF treatments is of keen interest, especially regarding protein-bound uremic toxins such as p-cresyl sulfate (PCs)^22^ and indoxyl sulfate (IS)^23^, poorly removed by conventional techniques^24,25^ due to their binding affinity.

To this end, we implemented for the first time an original in vitro set-up composed by discarded plasma exchanges from uremic patients and out-of-date erythrocytes as an inexpensive and filled uremic toxin precursor matrix aimed to evaluate the removal and adsorption properties of distinct commercial hemodialyzers (Sureflux-21UX by Nipro, CTA; BG-2.1U by Toray, PMMA; Fx-1000 Cordiax by Fresenius, PS and Polyflux-210H by Gambro, PA) submitted to HD and HDF through the quantification of total extracted and adsorbed mass. The understanding of removal and adsorption mechanisms of commercial hemodialyzers are of remarked interest to individualize and optimize the membrane and convective modality selection for each patient.

## Results

### Uremic toxin removal

The initial mass at the in vitro reservoir for each solute and treatment configuration is shown in Table 1. Similar values were obtained for every molecule in all treatment conditions and there were no significant differences for all the molecules except for protein-bound toxins. Moreover, the degradation of the implemented uremic matrix was reasonably slow as we found a slight decrease of less than 2 mg/L/month on the concentration levels for all molecules in the provided plasma exchanges.

**Table 1:** Mean ± standard deviation of the initial mass for each solute and treatment.

Treatment	Cr (mg)	$\beta _{2}$ (mg)	Myo (mg)	Pr (mg)	$\alpha _{1}$ (mg)	Alb (g)	PCs (mg)	IS (mg)
PMMA-HDF $(\hbox {n}=5)$	$45.0\pm 2.4$	$7.9\pm 0.3$	$152.4\pm 14.1$	$8.0\pm 0.3$	$79.0\pm 12.1$	$33.2\pm 0.7$	$14.7\pm 0.4$	$2.12\pm 0.08$
CTA-HDF $(\hbox {n}=3)$	$44.8\pm 1.3$	$7.6\pm 0.3$	$136.8\pm 14.3$	$7.7\pm 0.2$	$81.9\pm 2.1$	$31.7\pm 1.1$	$14.8\pm 0.3$	$2.13\pm 0.05$
PS-HDF $(\hbox {n}=4)$	$45.0\pm 2.0$	$7.8\pm 0.5$	$142.0\pm 6.6$	$8.3\pm 0.2$	$83.6\pm 19.2$	$32.3\pm 0.9$	$14.1\pm 0.1$	$2.06\pm 0.15$
PA-HDF $(\hbox {n}=4)$	$44.4\pm 1.0$	$8.1\pm 0.3$	$150.5\pm 3.2$	$7.9\pm 0.7$	$87.3\pm 11.7$	$32.4\pm 1.5$	$15.4\pm 0.5$	$2.00\pm 0.11$
PMMA-HD $(\hbox {n}=3)$	$44.4\pm 0.8$	$7.8\pm 0.2$	$150.1\pm 8.6$	$8.2\pm 0.5$	$70.0\pm 7.5$	$32.7\pm 0.8$	$14.3\pm 0.2$	$2.05\pm 0.03$
CTA-HD $(\hbox {n}=3)$	$40.4\pm 6.3$	$7.8\pm 0.1$	$142.3\pm 1.2$	$7.9\pm 1.0$	$71.4\pm 13.8$	$32.4\pm 1.3$	$14.9\pm 0.2$	$2.01\pm 0.11$
PS-HD $(\hbox {n}=3)$	$43.7\pm 1.5$	$7.6\pm 0.2$	$143.9\pm 4.9$	$7.7\pm 0.2$	$71.2\pm 12.3$	$32.1\pm 1.6$	$14.5\pm 0.6$	$2.07\pm 0.09$
PA-HD $(\hbox {n}=4)$	$39.6\pm 7.6$	$8.1\pm 0.1$	$144.5\pm 8.3$	$8.1\pm 0.3$	$77.0\pm 10.8$	$32.2\pm 1.7$	$15.1\pm 0.5$	$1.83\pm 0.08$
*p*	0.397	0.269	0.335	0.746	0.520	0.827	0.003	0.010

The number of replicates is shown in parenthesis. Statistical significance was obtained by means of one-way ANOVA test.

*Cr* creatinine, $\beta _{2}$ $\beta _{2}$-microglobulin, *Myo* myoglobin, *Pr* prolactin, $\alpha _{1}$ $\alpha _{1}$-microglobulin, *Alb* albumin, *PCs* total p-cresyl sulfate, *IS* total indoxyl sulfate.

**Figure 1:**
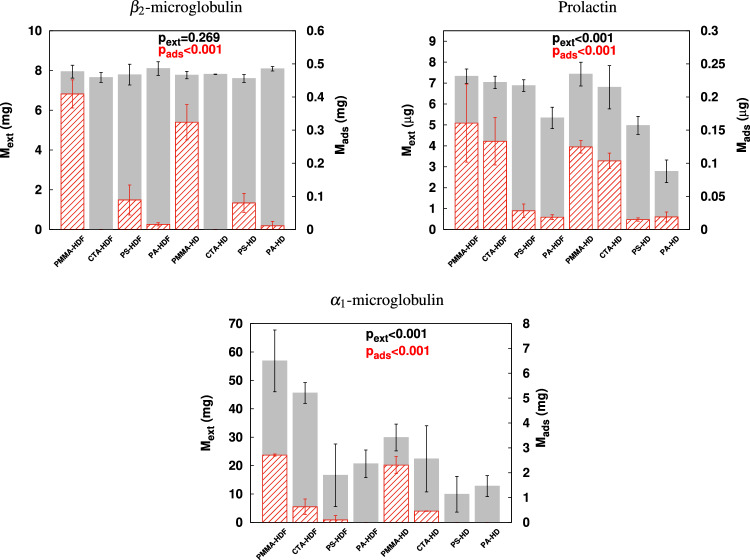
Mean ± standard deviation of the calculated extracted mass $M_{ext}$ (solid gray bars, left scale) and adsorbed mass $M_{ads}$ (patterned red bars, right scale) for every treatment condition. Top $p_{ext}$ and $p_{ads}$ are the overall significance values for $M_{ext}$ and $M_{ads}$ respectively by means of one-way ANOVA test. Statistical significance among treatments is not shown for better clarity and can be found as Supplementary Table S1 and S2.

The total extracted ($M_{ext}$) and adsorbed mass ($M_{ads}$) for the different solutes used in the tested hemodialyzers and treatment conditions are depicted in Fig. 1 whereas the concentration decay for each molecule and treatment is given as Supplementary Fig. S1. In general rule, a removal enhancement in the 17–30 kDa MW range due to convection was detected because all hemodialyzers obtained increased $M_{ext}$ values in HDF therapies compared to their performance in the non-convective mode. Moreover, the amount of extracted mass was significantly different ($p_{ext}<0.001$) among treatment conditions for solutes with MW over 17 kDa and remarked in the PMMA and CTA hemodialyzers. This behavior was clearly shown by the removal of $\alpha _{1}$-microglobulin in the convective modality, resulted in $M_{ext}=56.9\pm 10.9$ mg for PMMA-HDF ($p_{ext}<0.05$ vs. PMMA-HD, PS-HD and PS-HDF; $p_{ext}<0.01$ vs. PA-HD and PA-HDF) and $M_{ext}=45.6\pm 3.6$ mg for CTA-HDF ($p_{ext}<0.01$ vs. PA-HD and PA-HDF). For PCs and IS, similar removal patterns were obtained for all hemodialyzers tested regardless of the applied convection. For these latter solutes, the amount of $M_{ext}$ coincided with that to the initial mass as all post-treatment concentrations were below detection limits. Regarding adsorption profiles, all hemodialyzers obtained slightly increased $M_{ads}$ values for all molecules in the convective modality respect their performance in HD. However, the amount of adsorbed mass was tenfold or lower than $M_{ext}$ in all treatment conditions and molecules. Furthermore, the symmetrical and large pore hemodialyzers (i.e. PMMA and CTA) obtained the highest $M_{ads}$ values compared to the asymmetrical membranes (i.e. PS and PA) except for $\beta _{2}$-microglobulin. In this latter case, the adsorption properties of the PMMA hemodialyzer were confirmed as this particular membrane obtained the highest values compared to the other hemodialyzers tested, e.g., $M^{PMMA-HDF}_{ads}=0.41\pm 0.04$ mg ($p_{ads}<0.05$ vs. PA-HD, PA-HDF, CTA-HD and CTA-HDF; $p_{ads}<0.01$ vs. PS-HD and PS-HDF) and $M^{PMMA-HD}_{ads}=0.32\pm 0.05$ mg ($p_{ads}<0.05$ vs. all other filters and treatment modalities except PMMA-HDF). This hemodialyzer also showed the highest adsorption values for the other molecules analyzed. However, the amount of $\beta _{2}$-microglobulin $M_{ads}$ in CTA hemodialyzers was negligible, regardless of the chosen modality. Finally, creatinine and myoglobin adsorbed mass was unable to determine as a consequence of sample processing and detection threshold respectively. Likewise adsorbed levels of PCs and IS were indeterminable. The extracted mass is given as a Supplementary Fig. S2.

### Albumin removal

**Figure 2:**
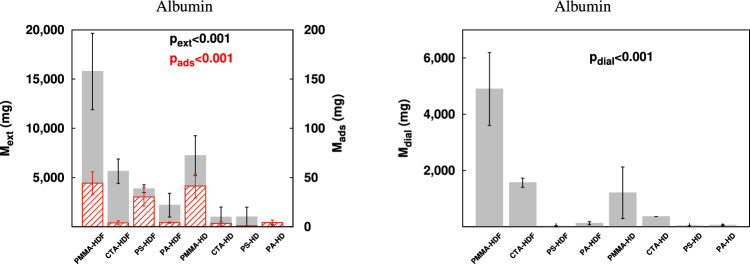
Mean ± standard deviation of albumin $M_{ext}$ (solid gray bars, left scale) and $M_{ads}$ (patterned red bars, right scale) both at the left and $M_{dial}$ (solid gray bars) at the right. $p_{dial}$ is the overall statistical significance by means of the one-way ANOVA test. The PMMA membrane showed substantial depuration in both treatment modalities whereas the albumin removal by the CTA membrane was enhanced by HDF. The amount of $M_{ads}$ for PS-HDF was notable.

Albumin depuration was assessed by means of the quantification of $M_{ext}$ and $M_{ads}$ (Fig. 2 left) and additionally by the amount of mass recovered in the dialysate, $M_{dial}$ (Fig. 2 right). This strategy allowed to relate the results to a complete transport description for this molecule. Thus, high values of $M_{ext}$ and $M_{dial}$ and reduced $M_{ads}$ would represent preferential non-adsorptive removal for this solute. For instance, the PMMA hemodialyzer obtained the highest $M_{ext}$ ($15.8\pm 3.9$ g), $M_{ads}$ ($44.3\pm 11.5$ mg) and $M_{dial}$ ($4.9\pm 1.3$ g) in the convective modality. However, in the particular case of the CTA membrane in HDF, values of $M_{ext}=5.6\pm 1.2$ g and $M_{dial}=1.6\pm 0.2$ g were obtained whereas adsorption mass was as low as $3.9\pm 2.1$ mg. The PS-HDF treatment reached values of $M_{ext}=3.9\pm 0.4$ g and $M_{ads}=30.4\pm 9.3$ mg but had negligible $M_{dial}=18\pm 18$ mg.

### Overall depuration

A global removal score, $\varphi$, as an overall depuration value over a wide range of MW solutes is summarized in Table 2. Mean values in the range 0.60–0.80 are expected for good removal efficiency in the MW range studied. However, lower values were found for PA-HD treatments as a consequence of moderated reduction ratio (*RR*) values for molecules with a MW higher than 12 kDa, i.e., $RR_{myoglobin}=0.48\pm 0.03$, $RR_{prolactin}=0.25\pm 0.08$ and $RR_{\alpha _{1}-microglobulin}=0.05\pm 0.08$. Furthermore, despite the significant *RR* for middle and high MW molecules obtained by PMMA-HDF treatment ($RR_{myoglobin}=0.99\pm 0.01$, $RR_{prolactin}=0.91\pm 0.01$ and $RR_{\alpha _{1}-microglobulin}=0.71\pm 0.05$), the strong penalization due to albumin removal ($RR_{albumin}=0.46\pm 0.12$) had a clear impact on the removal score, which resulted in the lowest value. The *RR* profile obtained for each treatment condition is given as Supplementary Fig. S3.

**Table 2:** Mean ± standard deviation of the global removal score, $\varphi$, for each treatment condition.

$\varphi$	PMMA	CTA	PS	PA
HD	$0.68\pm 0.05 ^{{\mathrm{a}}}$	$0.82\pm 0.02^{{\mathrm{a,c,d,e}}}$	$0.70\pm 0.03^{{\mathrm{a,b}}}$	$0.50\pm 0.09^{{\mathrm{b}}}$
HDF	$0.38\pm 0.08$	$0.75\pm 0.02 ^{{\mathrm{a}}}$	$0.73\pm 0.04 ^{{\mathrm{a}}}$	$0.72\pm 0.03 ^{{\mathrm{a,b}}}$

The penalization effect of albumin depuration is clearly seen in the lowest value obtained for PMMA in HDF treatments.${}^{\mathrm{a}}p<0.05$ versus PMMA-HDF, ${}^{\mathrm{b}}p<0.05$ versus CTA-HD, ${}^{\mathrm{c}}p<0.05$ versus PA-HDF, ${}^{\mathrm{d}}p<0.05$ versus PA-HD and ${}^{\mathrm{e}}p<0.05$ versus PS-HDF.

## Discussion

This study implemented successfully for the first time an innovative, inexpensive and blood equivalent physiological in vitro matrix filled with a plethora of distinct uremic toxins aimed to estimate and quantify the removal and adsorption properties of distinct material and structure high-flux hemodialyzers submitted to convective on-line hemodiafiltration.

The ongoing research in hemodialyzer technology has strongly focused on enhancing material biocompatibility and membrane design which may deliver different treatment outcomes. Thus, the fiber material, the configuration (plate or hollow fiber) and the structure of the capillary (sponge-like, finger-type, inner diameter, wall thickness, pore radius and density) have been continuously improved to deliver the most efficient renal replacement therapy. In addition, the conditions under hemodialyzers are used, i.e., blood and dialysate flows, ultrafiltration profiles and diffusive or convective modalities, may dramatically affect the performance.

The incorporation of high convective techniques proved to be clinically relevant in the depuration of middle and high MW solutes^26,27^. This depuration enhancement was highlighted by our in vitro procedures as each hemodialyzer increased the amount of extracted mass compared to its performance in the non-convective mode. This outcome was especially marked for the symmetric PMMA and CTA hemodialyzers. To this regard, we suspect that the aforementioned membranes with a large pore radius, $r_{p}$ (7 nm and 7.2 nm respectively) could ease the transport of solutes with a wider Stoke’s radius $r_{s}$, such as $\alpha _{1}$-microglobulin ($r_{s}=2.4$ nm^28^) and albumin ($r_{s}=3.6$ nm^29^). Hence, the moderated solute-to-pore radius ratio ($\frac{r_{s}}{r_{p}}\sim 0.5$) ensures optimal solute movement through the pore channel towards the dialysate side. Of note, however, the poor results for the PA hemodialyzer, despite the large pore size of the innermost layer. According to Hedayat et al.^30^, reduced porosity (i.e. less number of pores), rather than pore size, may affect the removal efficiency of this particular hemodialyzer in mid-range MW solutes.

Regarding adsorption mechanisms, we initially hypothesized that convection and the formation of a cell-free layer from the Fahraeus-Lindqvist effect could enhance the deposition of solutes at the inner wall of the membrane. Whilst the convective modality slightly enhanced $M_{ads}$, the obtained results were not substantially different for the same hemodialyzer when submitted to the non-convective modality. This behavior may be due to a prone surface-contact rather than a convective-related interaction. According to Rockel et al.^31^, a protein layer is formed mainly in the first 20–30 min of blood to membrane exposure. In our particular case, the performed 1-h experiments lasted enough to achieve a full protein layer regardless the transmembrane pressure applied. Thus, differences in the adsorbed mass due to convection were barely detected. Moreover, the specific adsorption potential of PA, PS and CTA membranes in HD were focused on essential proteins and immunoglobulins as obtained by several studies^32–35^. Our results are in total agreement with the data presented, among others, by Urbani et al.^36^, as we found that $\beta _{2}$-microglobulin was detected in greater quantities in PS membranes than in CTA membranes, whereas albumin was more present in CTA than in PS in the non-convective modality. We additionally expanded the adsorption behavior of molecules such as prolactin and $\alpha _{1}$-microglobulin towards the aforementioned membranes.

**Figure 3:**
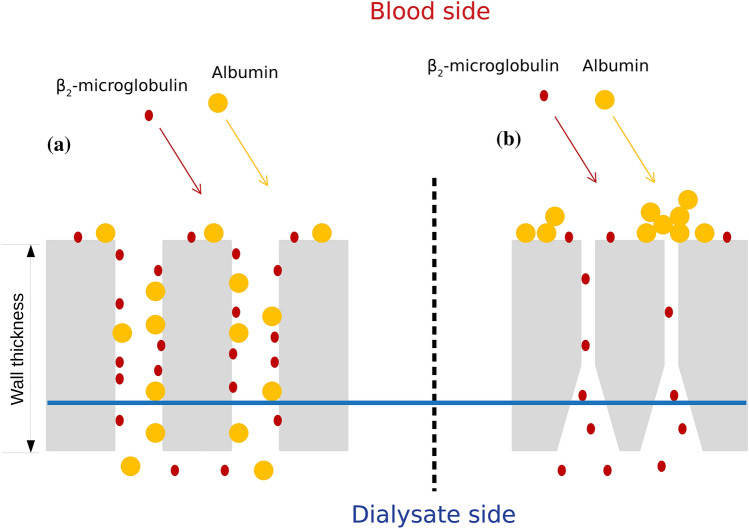
Schematic representation of adsorption regimens regarding structural configuration. A symmetric and large pore size membrane (**a**) may lead to a standard blocking model due to internal pore deposition. In an asymmetric and reduced pore size membrane (**b**) intermediate blocking or cake filtration laws could be the preferential mechanisms of adsorption for high-MW solutes, as these molecules tend to be deposited at the blood side of the pore channel.

Finally, we attempted to relate the adsorptive results obtained to simplified pore blocking models. According to our data, both the pore size and the wall structure and thickness may lead to a predominant pore-blocking behavior, which in turn is related to adsorption (Fig. 3). We hypothesize that symmetrical membrane frames (as for PMMA and CTA with 30 $\upmu \hbox {m}$ and 15 $\upmu \hbox {m}$ wall thickness respectively) and large pore sizes could allow middle and high MW molecules to enter the pore channel and be adsorbed along the capillary wall thickness. In these cases, the standard blocking model will be preferential and leads to pore constriction. Moreover, the increased $M_{ads}$ obtained for the PMMA membrane compared to CTA could be explained as the wall thickness of the former is twice that of the latter. However, the singular behavior of the PS membrane with low albumin depuration but significant adsorption in HDF could be attributed to the reduced innermost layer pore radius (3.3 nm) of the hemodialyzer. The high convection and the structural configuration lead to a solute-to-pore radius of $\frac{r_{s}}{r_{p}}\sim 1$. Under these conditions, the standard blocking model is no longer applicable and intermediate blocking and cake filtration laws may be predominant, reducing the number of pores available and increasing transport resistance, which in turn, enhances the adsorption at the capillary innermost layer. The above-exposed behavior could be in concordance with the mechanisms exposed by Wang and Tarabara in^37^ for membranes with a cut-off in the range of 30–100 KDa.

The present study has, nevertheless, several limitations. Our proposed in vitro procedures used a mixture of plasma exchanges from uremic patients and out-of-date erythrocytes. This reasonable stable matrix has not been reported previously in the literature but has provided similar results as other groups using bovine or healthy donor blood. However, we are concerned that the possible degradation and hemolysis of this matrix in an in vitro environment could have hampered some essential effects present in donor whole blood. Besides, the global removal score is presented as an overall depuration value for a wide MW uremic toxin range and in an in vitro performance. We are aware of the strong dependence of the selected solutes on performing the calculation. Further development of the proposed formula is therefore needed to obtain a validated score with clinical applicability. Moreover, the discussion related the obtained results to simplistic and straight pore models. This is a mere approximation as dialysis membranes are complex and intricate tortuous three-dimensional structures. In addition, solutes were considered as stand-alone solid spheres. The possible mechanisms of folding or de-folding, which in turn may affect their relative sizes and hindrance factors, were not considered. Likewise, we did not take into account the interaction among the different solutes or the importance of solute and membrane hydrophilicity or electric potential. Finally, the statistical power of the tests applied was low due to the limited number of replicates.

In conclusion, our proposed innovative mixture of plasma exchanges and out-of-date erythrocytes offers an inexpensive and adequate uremic matrix to successfully evaluate removed and adsorbed mass of several molecules in hemodialysis membranes. Our results reinforce that hemodiafiltration enhances middle and high MW removal but slightly increases adsorption, mainly due to a standard blocking pore model for symmetrical and large pore hemodialyzers. However, the significant loss of albumin in these latter membranes do not lead to enhanced protein-bound toxin removal. The similar cost-per-unit of the analyzed hemodialyzers may suggest the preferential use of asymmetric membranes in hemodiafiltration to attain a remarkable removal profile over a wide range of MW solutes and avoid protein loss.

## Methods

### Experimental set-up

The present study was approved by the Ethical Committee of Hospital Clínic (code HCB/2016/0057) and was conducted in accordance with the Declaration of Helsinki principles. Experiments were carried out in a novel uremic blood composed by an initial mixture of 625 mL of plasma exchanges from adult kidney ABOi transplant recipients (five subject pool), 500 mL of out-of-date ($\le$ 35 days) erythrocytes (final hematocrit $33\pm 3\%$) and 1000 UI sodium heparin which was stirred and maintained at $37\,^{\circ }\hbox {C}$ until being connected to a Fresenius Medical Care (FMC 5008) hemodialysis device. De-identified plasma exchanges, erythrocyte bags and informed consent from the subjects were obtained in cooperation with the local blood bank and the Departament de Hemoterapia i Hemostasia, Hospital Clínic de Barcelona. HD and HDF treatments were performed using commercial hemodialyzers (listed in Table 3) with the following parameters: blood flow (Qb) of 400 mL/min, dialysate flow (Qd) of 600 mL/min, ultrafiltration flow of 10 mL/h, substitution flow (Qi) of 134 mL/min (0 in HD), and treatment time (td) of 60 min.

**Table 3:** Characteristics of the tested hemodialyzers.

Characteristics	Filter commercial name			
	BG-2.1U (PMMA)	FX-1000 Cordiax (PS)	Sureflux-21UX (CTA)	Polyflux-210H (PA)
Main material	$\hbox {Polymethylmethacrylate}^{\mathrm{a}}$	$\hbox {Helixone plus}^{\mathrm{b}}$	Cellulose triacetate	$\hbox {Polyamix}^{\mathrm{c}}$
Structure	Symmetric	Asymmetric	Symmetric	Asymmetric
Inner diameter ($\upmu$m)	200	210	200	215
Wall thickness ($\upmu$m)	30	35	15	50
Av. blood-side pore radius (nm)	7	3.3	7.2	20
Kuf (mL/h/mmHg)	41	76	46	85
Area ($\mathrm{m}^{2}$)	2.1	2.3	2.1	2.1
S$_{\beta _{2}-microglobulin}$	n.d.	0.9	n.d.	0.82
S$_{myoglobin}$	n.d.	0.5	0.625	0.37

Their hydraulic permeability (Kuf), the total area and the reported sieving coefficients are also shown.

*n.d.* no available data.

${}^{\mathrm{a}}$Isotactic and syndiotactic polymethylmethacrylate polymer.

${}^{\mathrm{b}}$Polysulfone and polyvinylpyrrolidone blend.

${}^{\mathrm{c}}$Polyarylethersulfone, polyvinylpyrrolidone and polyamide blend.

Blood samples were drawn from the arterial port prior to treatment initiation (triggered by the FMC 5008 optical blood sensor) and at times $\hbox {t}=10'$, 20′ and 60′. Levels of creatinine, total p-cresyl and indoxyl sulfate, $\beta _{2}$-microglobulin, myoglobin, prolactin, $\alpha _{1}$-microglobulin and albumin were measured. A constant extraction of dialysate (rate 10 mL/min, total 60 mL collected) was performed to quantify albumin loss ($M_{dial}$). Treatment parameters displayed by the hemodialysis device at the end of each treatment such as the dialysis dose Kt (L), ionic dialysance K (mL/min), total blood processed $V_{blood}$ (L), convective volume $V_{sust}$ (L) and average transmembrane pressure (TMP, mmHg) were recorded (Supplementary Results Table S3).

### Elution protocol

**Figure 4:**
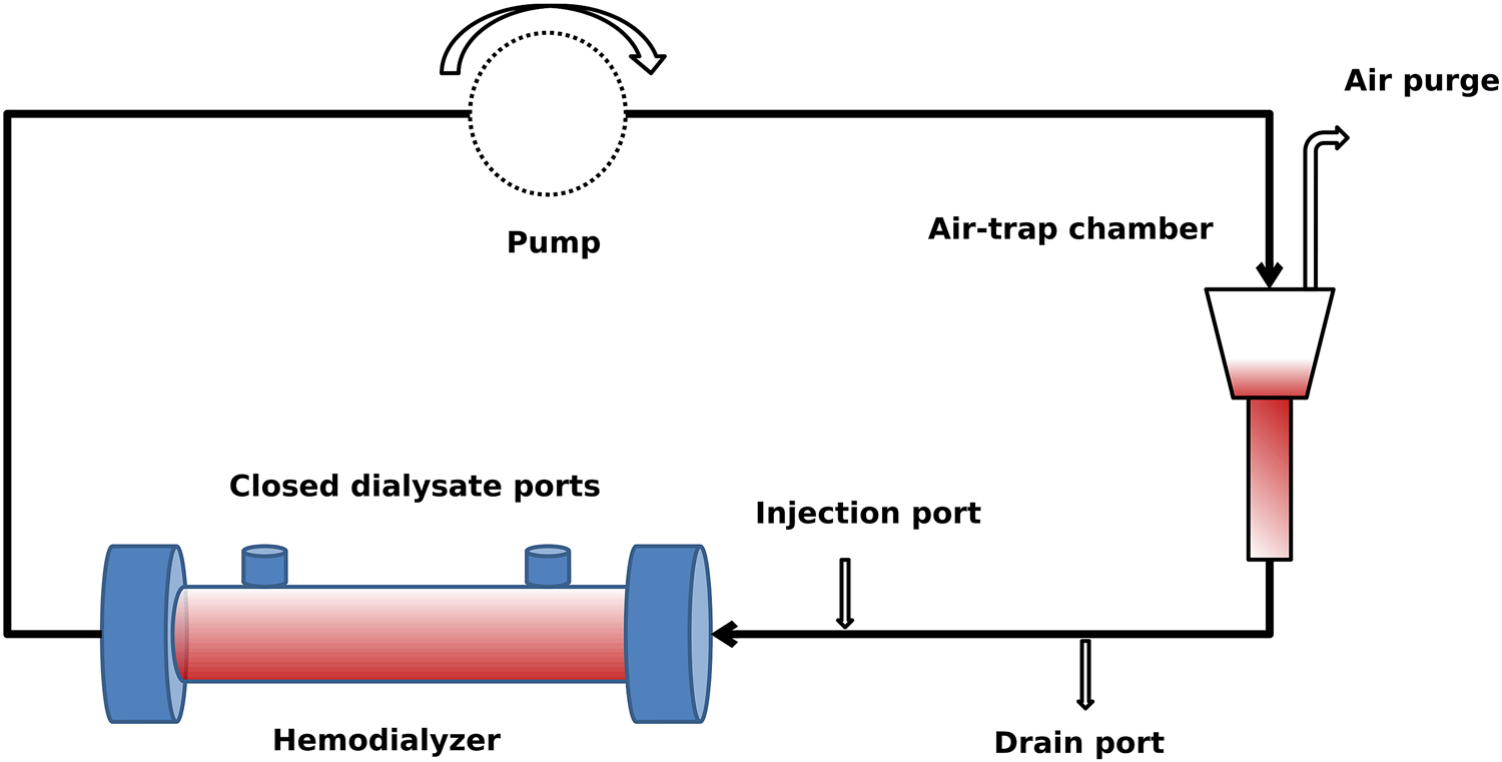
A closed circuit was implemented by the help of two 3-way keys (injection and drain ports) and a peristaltic Longerpump WT-600-2J to allow the introduction and extraction of the different solutions within the hemodialyzer fibers. The air-trap chamber was used exclusively to allow air removal from the circuit.

Once the hemodialysis treatment finished, an adapted elution protocol from Mares et al.^32^ was implemented. Firstly, 1 L of saline was single-passed at 30 mL/min to remove any residual blood. Afterwards, the dialyzer was placed into a closed circuit (Fig. 4) and 80 mL of PBS/EDTA (1$\times$/3 mM) was introduced and left at 96 mL/min for 30 min. Then, 80 mL of 40% v/v acetic acid was introduced while the previous mixture was discarded by the help of 3-way connectors and was left again at 96 mL/min for 30 minutes. The next step consisted in the addition of 80 mL of a strong chaotropic solution containing urea (7 M), Thiourea (2 M), CHAPS (4% v/v), DTT (120 mM) and TRIS-BASE (3 mM), while discarding the previous mixture. The new mixture was allowed to stand at 96 mL/min for 1 h. Lastly, 80 mL of PBS (1$\times$) was introduced to the circuit and all the obtained eluate ($V_{eluate}$, 160–250 mL) was collected into 50 mL polypropylene tubes and stored at $-\,80\,^{\circ }\hbox {C}$ until processing. To quantify the absorbed mass ($M_{ads}$) of each molecule, 40 mL of the obtained eluate was pre-filtered by a Filtropur 0.45 $\upmu \hbox {m}$ filter to remove further cellular debris and was concentrated by means of a centrifugal concentrator of 5 KDa cut-off membrane to a final volume ($V_{conc}$) of $\sim$ 2–4 mL. Finally, 1 mL of the obtained concentrate was assayed to obtain concentration levels ($C_{conc}$) of total p-cresyl and indoxyl sulfate, $\beta _{2}$-microglobulin, myoglobin, prolactin, $\alpha _{1}$-microglobulin and albumin.

### Hemodialyzer removal parameters

The *RR* for each solute was calculated by Equation 1 as

1 $$\begin{aligned} \begin{aligned} RR&=\frac{C(0)-C(t_{d})}{C(0)}&\qquad&if&\qquad&C(t_{d}) < C(0)\\ RR&= 0&\qquad&if&\qquad&C(t_{d}) \ge C(0), \end{aligned} \end{aligned}$$

where *C*(0) and $C(t_{d})$ are the concentration of each solute at the beginning and at the end of the treatment. Equation 1 avoids negative values of RR which would mean an increased post-treatment concentration due to the slightly ultrafiltered volume. In these particular cases, RR was assumed to be 0 (see also Eq. 4).

The extracted mass $M_{ext}$ for each solute was calculated by Equation 2 as

2 $$\begin{aligned} M_{ext}=V(0)C(0)-V(t_{d})C(t_{d}) \end{aligned}$$

with *V*(0) and $V(t_{d})$ being the volume of the reservoir at the beginning and at the end of the treatment, respectively. The term *V*(0)*C*(0) refers to the initial mass at the reservoir. The adsorbed mass $M_{ads}$ was therefore calculated as

3 $$\begin{aligned} M_{ads}=C_{conc}V_{conc}\dfrac{V_{eluate}}{40} \end{aligned}$$

Furthermore, a global removal score, $\varphi$, was calculated as

4 $$\begin{aligned} \begin{aligned} \varphi =&\dfrac{\sum \nolimits _{i=1}^{5} RR_{i}}{5}-RR_{albumin}&\qquad&for&\qquad&RR_{albumin}>0\\ \varphi =&\dfrac{\sum \nolimits _{i=1}^{5} RR_{i}}{5}&\qquad&for&\qquad&RR_{albumin}<0, \end{aligned} \end{aligned}$$

with *i* being the number of non-protein-bound uremic toxins considered, in our case five. This score, similar to the one presented by Maduell et al.^38^ represents an overall depuration value over a wide range of MW for each treatment type and emphasizes the albumin removal as a strong deleterious effect. If there were a perfect hemodialyzer, the total depuration of solutes and no reduction of albumin would lead to $\varphi =1$. In contrast, the worst possible scenario without toxin depuration and total depletion of albumin would lead to $\varphi =-1$. Under in vitro conditions, $\varphi$ values around 0.60–0.80 are to be expected.

### Statistical analysis

All variables are given by their mean and standard deviation. One-way ANOVA and post-hoc Games-Howell test for unbalance data was implemented, assuming $\alpha =0.05$ and $\beta =0.20$ to asses statistical significance among treatment conditions in all the studied variables using software package SPSS v.19.

### Supplementary information

Supplementary information.
