# Supplementary material for: Assessment of removal and adsorption enhancement of high-flux hemodialyzers in convective therapies by a novel in vitro uremic matrix
Source: Sci Rep. 2020 Oct 15;10:17403. doi: 10.1038/s41598-020-74528-5 (PMC7562951; doi:10.1038/s41598-020-74528-5)
Supplement: Supplementary file 1 — Supplementary information. [file 41598_2020_74528_MOESM1_ESM.pdf]

Supplementary Information

Assessment of removal and adsorption enhancement of high-flux hemodialyzers in convective therapies by a novel in vitro uremic matrix

Miquel Gomez, Elisenda Bañon-Maneus, Marta Arias-Guillén and Francisco Maduell

Prolactin	CTA-HDF	PS-HDF	PA-HDF	PMMA-HD	CTA-HD	PS-HD	PA-HD
PMMA-HDF	0.877	0.468	0.010	1.000	0.969	0.015	<0.001
CTA-HDF		0.993	0.027	0.929	1.000	0.026	0.001
PS-HDF			0.034	0.764	1.000	0.035	0.001
PA-HDF				0.052	0.498	0.952	0.005
PMMA-HD					0.962	0.036	0.002
CTA-HD						0.350	0.590
PS-HD							0.190
α_1-microglobulin							
PMMA-HDF	0.491	0.014	0.007	0.033	0.100	0.023	0.003
CTA-HDF		0.066	0.006	0.084	0.284	0.134	0.001
PS-HDF			0.994	0.495	0.994	0.962	0.994
PA-HDF				0.356	1.000	0.594	0.321
PMMA-HD					0.934	0.275	0.056
CTA-HD						0.762	0.824
PS-HD							0.992
Albumin-dialysate							
PMMA-HDF	0.189	0.097	0.100	0.013	0.111	0.097	0.098
CTA-HDF		0.015	0.010	0.990	0.026	0.016	0.015
PS-HDF			0.281	0.537	0.004	0.699	0.398
PA-HDF				0.598	0.074	0.385	0.532
PMMA-HD					0.747	0.546	0.556
CTA-HD						-	0.005
PS-HD							0.699

Supplementary Results Table S1. Games-Howell post-hoc statistical significance of the extracted mass, M_{ext}, among different treatment conditions for each solute.

β_2-microglobulin	CTA-HDF	PS-HDF	PA-HDF	PMMA-HD	CTA-HD	PS-HD	PA-HD
PMMA-HDF	0.016	0.003	0.016	0.501	0.016	0.005	0.010
CTA-HDF		0.163	0.125	0.039	-	0.167	0.782
PS-HDF			0.251	0.028	0.163	1.000	0.218
PA-HDF				0.041	0.125	0.238	0.997
PMMA-HD					0.039	0.035	0.033
CTA-HD						0.167	0.782
PS-HD							0.193
Prolactin							
PMMA-HDF	0.993	0.464	0.438	0.955	0.833	0.429	0.438
CTA-HDF		0.141	0.128	1.000	0.845	0.122	0.122
PS-HDF			0.658	0.024	0.082	0.388	0.828
PA-HDF				0.073	0.130	0.831	1.000
PMMA-HD					0.612	0.084	0.032
CTA-HD						0.136	0.084
PS-HD							0.964
α_1-microglobulin							
PMMA-HDF	0.163	0.004	<0.001	0.753	<0.001	<0.001	<0.001
CTA-HDF		0.584	0.508	0.153	0.963	0.508	0.508
PS-HDF			0.937	0.125	0.279	0.937	0.937
PA-HDF				0.169	0.031	-	-
PMMA-HD					0.208	0.169	0.169
CTA-HD						0.031	0.031
PS-HD							-
Albumin							
PMMA-HDF	0.103	0.727	0.110	1.000	0.102	0.097	0.099
CTA-HDF		0.151	1.000	0.353	1.000	0.505	1.000
PS-HDF			0.163	0.914	0.148	0.135	0.149
PA-HDF				0.360	0.987	0.076	1.000
PMMA-HD					0.349	0.335	0.351
CTA-HD						0.531	0.997
PS-HD							0.692

Supplementary Results Table S2. Games-Howell post-hoc statistical significance of the adsorbed mass, M_{ads}, among different treatment conditions for each solute.

Treatment	Kt (L)	K (mL/min)	V_{blood} (L)	V_{sust} (L)	TMP (mmHg)
PMMA-HDF (N=5)	15.2 ± 0.6	247.8 ± 10.6	23.1 ± 0.1	7.4 ± 0.0	203.0 ± 9.2
CTA-HDF (N=3)	15.8 ± 0.4	254.3 ± 5.9	23.5 ± 0.2	7.5 ± 0.1	133.3 ± 2.9
PS-HDF (N=4)	16.1 ± 0.2	254.7 ± 7.6	23.3 ± 0.1	7.5 ± 0.1	118.3 ± 7.6
PA-HDF (N=4)	15.6 ± 0.9	251.3 ± 14.5	23.5 ± 0.2	7.1 ± 0.6	76.3 ± 4.8
PMMA-HD (N=3)	7.4 ± 0.1	122.0 ± 1.0	23.5 ± 0.2	0.0	20.0 ± 0.0
CTA-HD (N=3)	7.1 ± 0.4	114.0 ± 5.6	23.9 ± 0.5	0.0	25.0 ± 5.0
PS-HD (N=3)	7.6 ± 0.1	123.3 ± 3.8	23.6 ± 0.6	0.0	20.0 ± 0.0
PA-HD (N=4)	8.1 ± 0.1	131.5 ± 4.2	23.2 ± 0.4	0.0	20.0 ± 0.0
p	<0.001	<0.001	0.071	<0.001	<0.001

Supplementary Results Table S3. Mean ± standard deviation of dialysis dose (Kt), ionic dialysance (K), total blood processed (V_{blood}), convective volume (V_{sust}) and transmembrane pressure (TMP) for the different conditions tested. The number of replicates is shown in parenthesis. Statistical significance was obtained by the one-way ANOVA test. The removal enhancement due to convective transport is translated in an overall relative increase of K and Kt of 108% and 106% respectively, whilst the total amount of blood processed was nearly the same for all conditions. The lower TMP values obtained for PA-HDF treatments compared to the other convective modalities could be related to the large fiber inner diameter (215 μm) for this membrane.

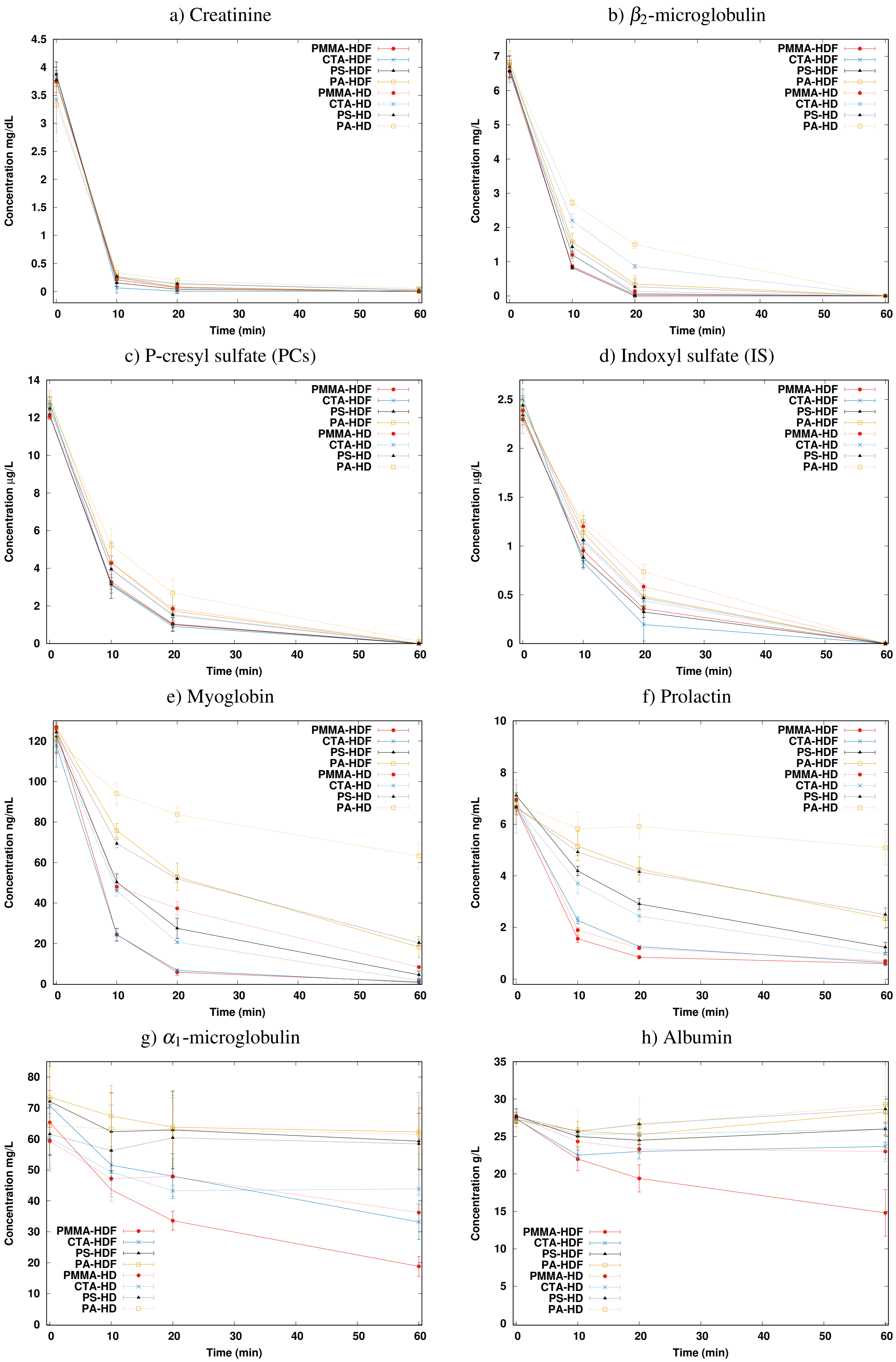

Supplementary Results Figure S1. Mean and standard deviation (error bars) of concentration decay over time for each molecule and treatment configuration. Distinct removal profiles are evident for all solutes except creatinine.

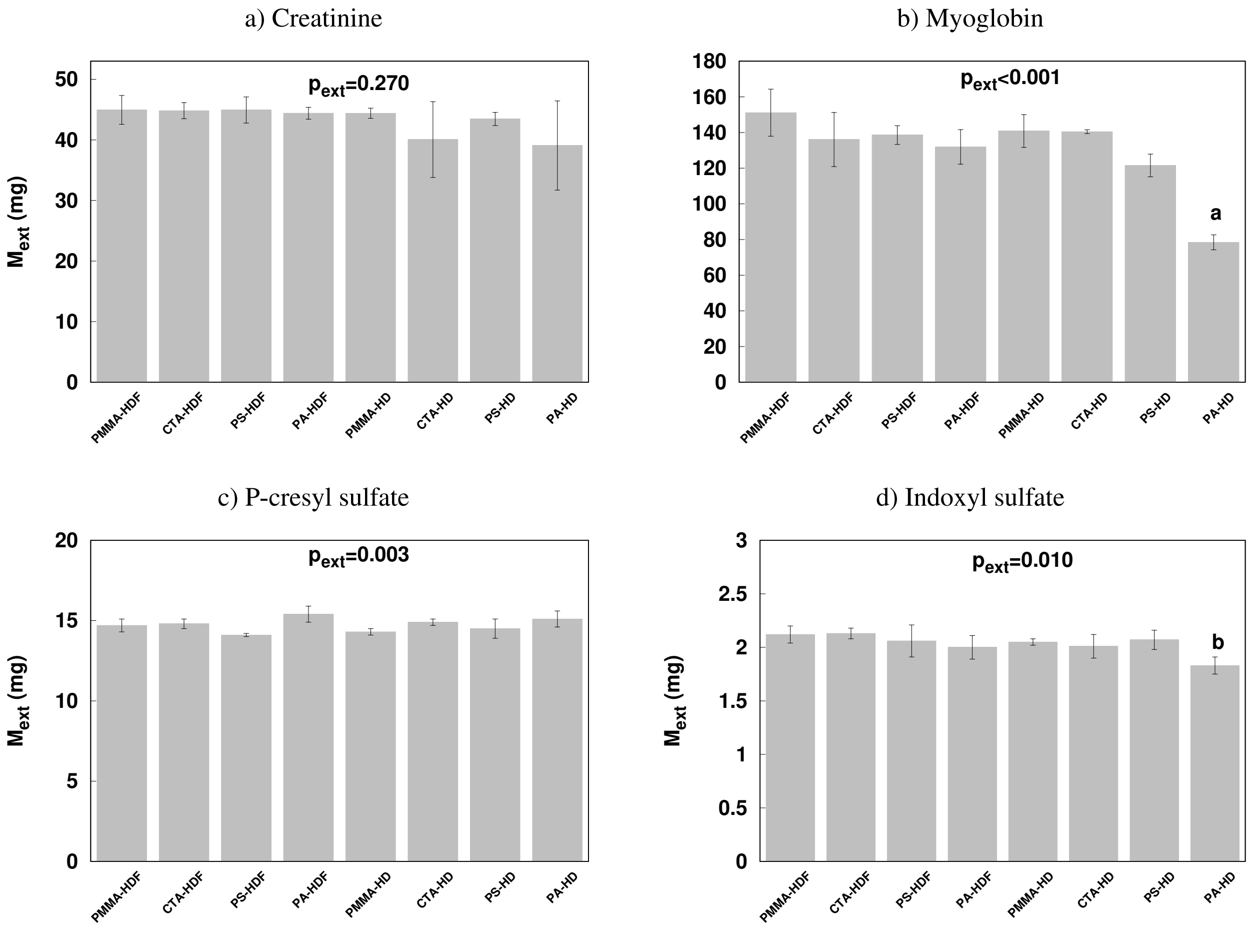

Supplementary Results Figure S2. Mean and standard deviation (error bars) of the calculated extracted mass M_{ext} for creatinine, myoglobin, total p-cresyl sulfate (PCs) and total indoxyl sulfate (IS). The amount of adsorbed mass for creatinine was unable to determine as a consequence of the experimental processing of the samples. In the case of myoglobin, M_{ads} was below the detection limits in all treatment conditions. For PCs and IS, levels of M_{ads} were unable to obtain. Overall statistical significance between treatments is shown as p_{ext}. ^{a}p<0.05 vs. all other conditions; ^{b}p<0.05 vs. PMMA-HDF and CTA-HDF Games-Howell post-hoc analysis.

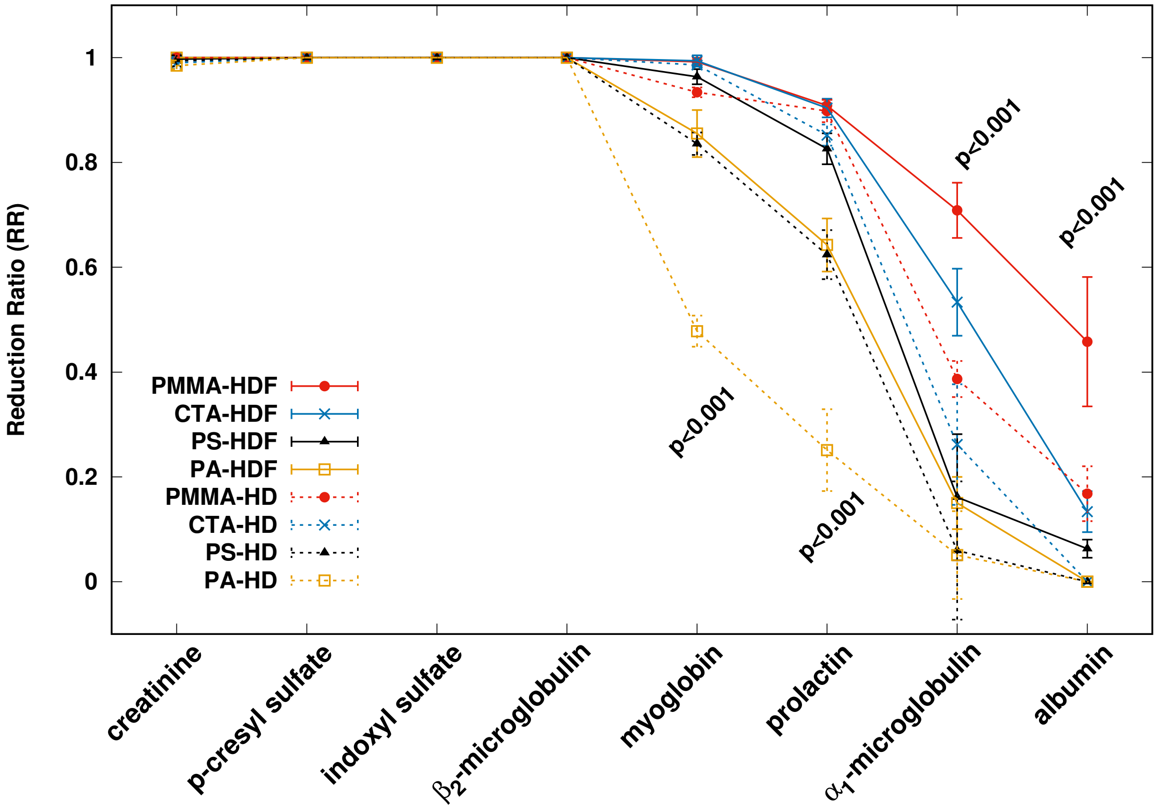

Supplementary Results Figure S3. Mean and standard deviation (error bars) for the reduction ratio (RR) for every hemodialyzer and treatment condition. Overall statistical significance among treatments is evident for molecules with a higher MW than β_2-microglobulin. Symmetrical PMMA and CTA hemodialyzers showed remarkable removal profiles over the range of uremic toxins analyzed despite non-negligible albumin depuration in HDF. The poor depurative efficiency of the PA membrane is demonstrated as only reached a decent removal profile when submitted to HDF.
